# Urban Dust Microbiome: Impact on Later Atopy and Wheezing

**DOI:** 10.1289/EHP158

**Published:** 2016-05-27

**Authors:** Christina Tischer, Fabian Weikl, Alexander J. Probst, Marie Standl, Joachim Heinrich, Karin Pritsch

**Affiliations:** 1 Institute of Epidemiology I, Helmholtz Zentrum München–German Research Center for Environmental Health, Neuherberg, Germany; 2 ISGlobal, Centre for Research in Environmental Epidemiology (CREAL), Barcelona, Spain; 3 Universitat Pompeu Fabra (UPF), Barcelona, Spain; 4 CIBER Epidemiología y Salud Pública (CIBERESP), Spain; 5 Institute of Biochemical Plant Pathology, Helmholtz Zentrum München–German Research Center for Environmental Health, Neuherberg, Germany; 6 Department of Earth and Planetary Sciences, University of California, Berkeley, Berkeley, California, USA; 7 Institute and Outpatient Clinic for Occupational, Social, and Environmental Medicine, Ludwig Maximilians University Munich, Munich, Germany

## Abstract

**Background:**

Investigations in urban areas have just begun to explore how the indoor dust microbiome may affect the pathogenesis of asthma and allery.

**Objective:**

We aimed to investigate the early fungal and bacterial microbiome in house dust with allergic sensitization and wheezing later in childhood.

**Methods:**

Individual dust samples from 189 homes of the LISAplus birth cohort study were collected shortly after birth from living room floors and profiled for fungal and bacterial microbiome. Fungal and bacterial diversity was assessed with terminal restriction fragment length polymorphism (tRFLP) and defined by Simpson’s Diversity Index. Information on wheezing outcomes and covariates until the age of 10 years was obtained by parent questionnaires. Information on specific allergic sensitization was available at child’s age 6 and 10 years. Logistic regression and general estimation equation (GEE) models were used to examine the relationship between microbial diversity and health outcomes.

**Results:**

Adjusted logistic regression analyses revealed a significantly reduced risk of developing sensitization to aero-allergens at 6 years and ever wheezing until the age of 10 years for exposure to higher fungal diversity [adjusted odds ratio (aOR) = 0.26 (95% CI: 0.10, 0.70), and 0.42 (95% CI: 0.18, 0.96), respectively]. The associations were attenuated for the longitudinal analyses (GEE) until the age of 10 years. There was no association between higher exposure to bacterial diversity and the tested health outcomes.

**Conclusion:**

Higher early exposure to fungal diversity might help to prevent a child from developing sensitization to aero-allergens in early childhood, but the reasons for attenuated effects in later childhood require further prospective studies.

**Citation:**

Tischer C, Weikl F, Probst AJ, Standl M, Heinrich J, Pritsch K. 2016. Urban dust microbiome: impact on later atopy and wheezing. Environ Health Perspect 124:1919–1923; http://dx.doi.org/10.1289/EHP158

## Introduction

Farm environment has been considered the strongest protective effect in relation to asthma and allergy in children (von Mutius and Vercelli 2010). Studies on the mechanisms observed higher levels of endotoxin (bacterial lipopolysaccharide of Gram-negative bacteria), but also mold-related components including 1,3-β-d-glucan, a cell-wall component of most fungi and fungal extracellular polysaccharides (EPS) from the genera *Penicillium* and *Aspergillus* within settled dust in farming households (Karvonen et al. 2012; Schram-Bijkerk et al. 2005). It has been suggested that increased exposure might be partly responsible for the observed inverse associations (Braun-Fahrländer et al. 2002) through immunomodulatory effects (Schuijs et al. 2015). Evidence regarding the microbiome composition in dust from urban environments and its influence on the occurrence and development of allergic diseases is still scarce, and comprehensive understanding is lacking.

The microbial profile in urban environments might differ considerably from those in rural areas in levels, composition, and diversity (Pakarinen et al. 2008) and therefore might also have different effects on atopic outcomes. Until now, the assessment of the urban microbiome in dust has been only considered in a few studies. Those had small sample sizes (not exceeding 100 subjects) or were studies mainly focused on exposure assessment rather than on health outcomes (Adams et al. 2013a, 2013b, 2014; Barberán et al. 2015; Dannemiller et al. 2014, 2016; Kembel et al. 2012; Lynch et al. 2014). Moreover, to conclude on the impact of early exposure to the urban dust microbiome in relation to health outcomes in later childhood, cohort studies with a prospective study design and appropriate analyses methods are required.

In the present study, we investigated the diversity of the fungal and bacterial microbiome in dust from a population-based birth cohort from the city of Munich, Germany. We aimed to study the hypothesis whether early life exposure to fungal and bacterial diversity is associated with a decreased risk of allergic sensitization and wheezing later in childhood. These outcomes are major risk factors for asthma and allergic diseases.

## Materials and Methods

### Study Overview and Participants

LISAplus (the influence of life-style factors on the development of the immune system and allergies in East and West Germany plus the influence of traffic emissions and genetics study) is an ongoing birth cohort study with four research centers in Germany (Munich, Leipzig, Bad Honnef, and Wesel). Screening, recruitment, and exclusion criteria have been described in detail elsewhere (Heinrich et al. 2002; Zutavern et al. 2006). In short, a total of 3,094 healthy full-term neonates were recruited between December 1997 and January 1999. Only healthy full-term neonates with a gestational age ≥ 37 weeks were included in the study. The present analysis is based on a subgroup of children from the Munich study center with an available dust sample from the living room floor obtained at 3 months of age and follow-up information on outcomes until 10 years of age (*n* = 189). In the final models, the sample size varied between 110 and 189 subjects, depending on the included variables. Informed consent of the parents has been obtained from all participating subjects. The study was approved by the local ethics committee (the Bavarian Board of Physicians, reference number 01212).

### Assessment of Health Outcomes

The subjects were tested for specific sensitization at 6 and 10 years. Specific allergic sensitization was defined as a positive response (> 0.35 kU/L) to the “SX1 aero-allergen mixture” [timothy, rye, mugwort, mite (*Dermatophagoides pteronyssinus*), cat, dog and mold (*Cladosporium herbarum*) allergens]. Wheezing in the preceding 12 months was obtained at age of 6, 12, 18, 24 months and at 4, 6, and 10 years of age. Controls were defined as “complete” controls and had information available for all time points.

### Dust Sampling and Assessment of the Fungal and Bacterial Microbiome

**Figure 1:**
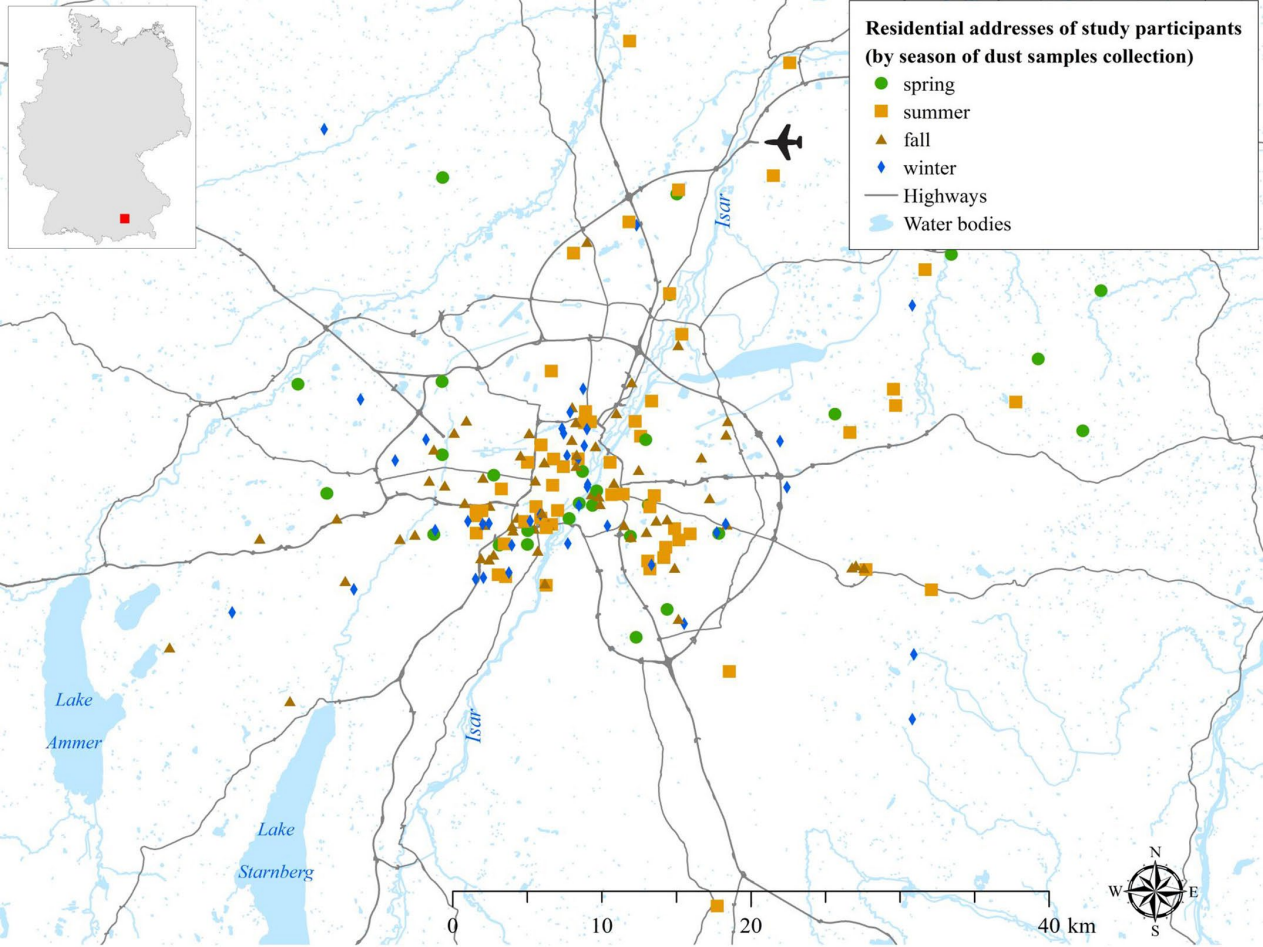
Map of the Munich urban area. The map covers the Larger Urban Zone of Munich and includes the position of 189 sampled households located within a radius of 37.5 km from the center of Munich. [Map created with ArcGIS 10.3 Geographical Information System (GIS) (ESRI, Redlands, CA).]

Settled dust samples from living room floors in the area of Munich (radius, 37.5 km; Figure 1) were obtained by trained inspectors when the children were 2–3 months old by using vacuum cleaners (Phillips, Hamburg, Germany) equipped with ALK filter holders (ALK, Hørsholm, Denmark) containing a paper filter. The sampling was done by vacuuming 1 m^2^ for 2 min for textile surfaces or 4 m^2^ for 4 min for smooth floors. The sampling period lasted 301 days. The samples were stored below –20°C. A detailed description of the dust sampling and analysis procedures has been published previously (Casas et al. 2013; Heinrich et al. 2002).

Frozen filter boxes with vacuumed dust were equilibrated to ambient conditions in a clean PCR (polymerase chain reaction) chamber with deactivated airflow for 60 min. Dust was released from the filter boxes, freed from obvious extraction obstacles (e.g., stones), and 100 mg were used to extract DNA with a PowerSoil-htp96 Soil DNA Isolation Kit (Mo-Bio Laboratories, Carlsbad, CA, USA). For tRFLP (terminal restriction fragment length polymorphism) DNA fingerprinting, DNA was PCR-amplified using a TopTaq DNA polymerase kit (Qiagen, Hilden, Germany) with primers ITS1F (5´-CTT​GGT​CAT​TTA​GAG​GAA​GTA​A-3´) (Gardes and Bruns 1993) and ITS4 (5´-TCC​TCC​GCT​TAT​TGA​TAT​GC-3´) (White et al. 1990) for fungal ITS (internal transcribed spacer) DNA with a mean amplicon length of 600 base-pairs (bp) (UNITE 2015). Most amplicons expected between 540 and 800 bp, or Bac27f (5´-AGA​GTT​TGA​TCM​TGG​CTC​AG-3´) (Jiang et al. 2006) and 907r (5-CCG​TCA​ATT​CMT​TTG​AGT​TT-3) (Mühling et al. 2008) for bacterial 16S rRNA genes (880 base-pairs amplicon). Forward primers were labeled with 6-FAM and reverse primers with 6-HEX fluorescent dyes, respectively. PCR profiles were done as follows: 4 min 94°C; 32 cycles of 60 sec 94°C, 60 sec 50°C, 90 sec 72°C; 5 min 72°C (fungi); and 5 min 94°C; 30 cycles of 45 sec 94°C, 45 sec 59°C, 45 sec 72°C; 5 min 72°C (bacteria). Products from two PCR reactions were pooled, purified, and digested with restriction enzyme HpyCH4IV (fungi) or MspI (bacteria). HpyCH4IV was chosen because of its performance in separating all fungal strains with ITS sequences available in the International Nucleotide Sequence Database (INSD) in a study by Alvarado and Manjón (2009). A similar performance in indoor dust was reconfirmed with an *in silico* restriction analysis of 91 ITS sequences from 38 fungal genera (Ascomycota, Basidiomycota, Zygomycota) using REPK v1.3 (Collins and Rocap 2007). The sequences were selected based on availability (INSD release 09/2012) and their description as commonly found in indoor environments, such as those found in the study by Pitkäranta et al. (2008) (data from a reference building; strains > 0.8% of total retrieved clones were considered). Sequences were obtained as described by Alvarado and Manjón (2009). Cleaned fragments were transferred to HiDi Formamid (Applied Biosystems, Foster City, CA, USA) containing MapMarker 1000-ROX (1:400; Bioventures, Murfreesboro, TN, USA) and separated with an ABI 3730 capillary sequencer (Applied Biosystems). Raw fragment tables were built with peak-scanner 2.0 (Applied Biosystems). T-REX v1.14 (Culman et al. 2009) was used for noise filtering with algorithms of Abdo et al. (2006) (SD multiplier 1) and for binning, alignment, and accounting of T-RF drift with the approach from Smith et al. (2005) (threshold 1 bp). For all analysis steps, data based on peak height instead of peak area was used following suggestions by Culman et al. (2008). Contamination was controlled with samples consisting of material scratched off from empty dust filters, and with non-template controls during PCR. The study population (*n* = 189) encompassed only samples of which DNA had been successfully amplified and electropherograms had passed peak scanner’s initial quality test (90% of 209 samples).

### Fungal and Bacterial Diversity in Dust

To assess possible relationships between the fungal and the bacterial microbiome in dust with later health outcomes, we determined the relative diversity (dependent on method and marker gene) of the fungal and bacterial microbiome. The microbial diversity can be assessed by taking into account species richness (the number of different kinds of species) and species evenness (a measure of the relative abundance of difference species). For the current investigation we used Simpson’s Diversity Index (Simpson 1949) as a measure for microbial diversity. The valuation of Simpson’s Diversity Index (expressed as 1-D) ranges from 0 (no diversity, all individuals belong to the same species) to 1 (maximum diversity). For doing so, OTU (operational taxonomic unit) abundances were rarefied (function “Rarefy”) to the lowest amount of signal present in the samples and the Simpson index calculated for each sample [vegan (Oksanen et al. 2013) and GUniFrac package (Chen 2012) in R (version 3.1.3; R Project for Statistical Computing)]. This step was repeated 1,000 times and averaged. Results were calculated separately for forward and reverse terminal restriction fragments (including labeled forward or reverse primers) and averaged.

### Statistical Analysis

To investigate possible relationships between exposure to fungal and bacterial diversity (Simpson index) with later allergic sensitization and wheezing outcomes, logistic regression and general estimation equation (GEE) models (logit link and exchangeable correlation structure) were used with the exposure (fungal and bacterial diversity) expressed in tertiles. Apart from the main analyses, we evaluated the association between exposure to microbial diversity and wheezing at earlier time points in childhood in sensitivity analyses. Exposure to fungal and bacterial diversity was categorized into tertiles for the analyses because not all functional relationships between exposure and health outcomes appeared to be linear. The logistic regression and GEE models have been adjusted for sex, maternal education, and season of dust sampling. All results are presented as adjusted odds ratio (aOR) with corresponding 95% confidence interval (CI). Statistical analyses were performed using the R programming environment (version 3.1.3; R Project for Statistical Computing).

## Results

The study population characteristics are depicted in Table 1. About two-thirds of the mothers (68%) held a high educational level compared with mothers with low or medium education (32%). During the months in summer and autumn, more dust samples were obtained than in winter and spring. There was very weak correlation between fungal and bacterial diversity (Spearman’s rho = –0.05). At the 6-year follow-up, 27% of the children were sensitized to aero-allergens, with 40% at 10 years. Ever wheezing at the age of 10 years was reported for 43% of the children.

**Table 1:** Characteristics of the study population.

Characteristic	*n*/*N* (%)
Study population	189
Female	87/189 (46)
Maternal education
Low and medium	60/187 (32)
High	127/187 (68)
Season of dust sampling
Winter	41/189 (22)
Spring	29/189 (15)
Summer	57/189 (30)
Autumn	62/189 (33)
Fungal diversity^*a*^
1st tertile	(0.403–0.863)
2nd tertile	(0.863–0.931)
3rd tertile	(0.931–0.977)
Bacterial diversity^*a*^
1st tertile	(0.179–0.693)
2nd tertile	(0.693–0.835)
3rd tertile	(0.835–0.941)
IgE aeroallergens (≥ 0.35 kU/L)—6 years	43/159 (27)
IgE aeroallergens (≥ 0.35 kU/L)—10 years	56/141 (40)
Wheezing ever (at 10 years)	73/170 (43)
^***a***^Ranges of the tertiles.	

### Regression Analyses

Table 2 shows that there was a statistically significant inverse association between higher exposure to fungal diversity around birth and sensitization to aero-allergens at 6 years (3rd tertile versus 1st tertile: aOR = 0.26; 95% CI: 0.10, 0.70), adjusted for covariates. High fungal diversity in dust also conferred protection for ever wheezing until the age of 10 years (3rd tertile versus 1st tertile: aOR = 0.42; 95% CI: 0.18, 0.96). However, in the longitudinal view (GEE models), considering the impact of several follow-ups and their correlation with each other, the inverse effects attenuated. The association between exposure to higher fungal diversity and sensitization to aero-allergens as well as wheezing did not attain statistical significance (aOR = 0.61; 95% CI: 0.24, 1.59, and aOR = 0.57; 95% CI: 0.26, 1.22, respectively). Sensitivity analyses revealed that there was also a statistically significant association with wheezing until 2 years in the logistic regression model as well as the GEE model for early exposure to fungal diversity (see Table S1). There was no significant association between exposure to bacterial diversity with any of the outcomes tested.

**Table 2:** Crude and adjusted*^a^* odds ratios (95% CIs) for the association between fungal and bacterial diversity (Simpson index, tertiles) and health outcomes.

Model	Crude analyses		Adjusted^*a*^ analyses	
	Fungal diversity	Bacterial diversity	Fungal diversity	Bacterial diversity
Logisitic regression models
Sensitization to aero-allergens (6 years)			*n* = 157	*n* = 157
2nd tertile vs. 1st tertile	0.79 (0.35, 1.80)	0.63 (0.27, 1.47)	0.66 (0.28, 1.56)	0.56 (0.23, 1.33)
3rd tertile vs. 1st tertile	0.31 (0.12, 0.79)	0.53 (0.22, 1.24)	0.26 (0.10, 0.70)	0.45 (0.18, 1.11)
Sensitization to aero-allergens (10 years)			*n* = 140	*n* = 140
2nd tertile vs. 1st tertile	1.41 (0.61, 3.22)	0.71 (0.31, 1.58)	1.13 (0.47, 2.70)	0.59 (0.25, 1.38)
3rd tertile vs. 1st tertile	1.32 (0.57, 3.07)	0.63 (0.27, 1.45)	1.01 (0.41, 2.51)	0.45 (0.18, 1.11)
Wheezing ever (10 years)			*n* = 168	*n* = 168
2nd tertile vs. 1st tertile	0.61 (0.29, 1.28)	0.62 (0.29, 1.33)	0.59 (0.27, 1.28)	0.60 (0.28, 1.30)
3rd tertile vs. 1st tertile	0.45 (0.21, 0.95)	1.00 (0.48, 2.07)	0.42 (0.18, 0.96)	1.00 (0.45, 2.06)
GEE models (longitudinal analysis)
Sensitization to aero-allergens until 10 years			*n* = 110	*n* = 110
2nd tertile vs. 1st tertile	1.04 (0.51, 2.12)	0.58 (0.29, 1.19)	0.89 (0.34, 2.32)	0.79 (0.33, 1.90)
3rd tertile vs. 1st tertile	0.69 (0.33, 1.41)	0.51 (0.25, 1.04)	0.61 (0.24, 1.59)	0.56 (0.22, 1.42)
Wheezing until 10 years			*n* = 159	*n* = 159
2nd tertile vs. 1st tertile	0.83 (0.45, 1.54)	0.81 (0.41, 1.57)	0.78 (0.40, 1.51)	0.74 (0.36, 1.53)
3rd tertile vs. 1st tertile	0.50 (0.26, 0.98)	1.14 (0.62, 2.09)	0.57 (0.26, 1.22)	0.98 (0.52, 1.84)
^a^Adjusted for sex, maternal education, and season of dust sampling.				

## Discussion

To the best of our knowledge, the present prospective study is the first that specifically considered the fungal and the bacterial microbiome in > 100 households in an urban environment. We observed that a higher exposure to fungal diversity in house dust around birth was significantly inversely related to aero-allergen sensitization status at 6 years as well as ever wheezing until the age of 10 years. However, considering several follow-up time points during the study period, the magnitude of the effects attenuated and the association did not attain statistical significance in the longitudinal view.

For farm and rural environments, studies have shown that early microbial exposure seems to be crucial for non-allergic immune response later in childhood and adulthood. Ege et al. (2011) observed in a cross-sectional analysis that children growing up on farms were exposed to a greater diversity of fungal and bacterial species, resulting in a lower prevalence of childhood asthma and atopy. As against farm studies, investigations in urban areas have just begun to explore how the indoor dust microbiome may affect the pathogenesis of asthma and allergic diseases. The CHAMACOS (Center for the Health Assessment of Mothers and Children of Salinas) birth cohort study in California (USA), used next-generation DNA sequencing of fungal ITS regions describing the fungal microbiome in settled house dust collected at 12 months of age. In this small case–control study (13 asthma cases and 28 controls), it has been observed that the asthma risk at 7 years of age was significantly increased for lower fungal diversity in dust within the first year of life (Dannemiller et al. 2014). One birth cohort study (URECA; Urban Environment and Childhood Asthma) across 104 children residing in an exclusively urban environment investigated the association of combined early-life exposure to allergens and bacteria on wheezing and atopic outcomes (Lynch et al. 2014). Lynch and colleagues observed that both exposure to high levels of allergen and a certain subset of bacteria taxa decreased the risk of allergic sensitization and wheezing outcomes at the age of 3 years. Our present study partly confirms what has been found recently; however, a unique feature of our study is the long follow-up period until later childhood. Although we also observed inverse associations of higher microbial exposure in relation to allergic sensitization and wheezing, the effects were significant only for higher fungal diversity and only for early childhood (6 years) but not at later age (10 years). As of today, no study in an urban setting could confirm protective effects of higher microbial exposure in relation to atopic outcomes until later childhood or young adulthood. We suggest that a possible reason might be that with increasing age, the school environment and activities conducted in different places might start to become more important, and the daily individual microbial exposure may change in composition and relevance (Tischer et al. 2015).

The present study has important strengths, such as a prospective study design, a larger sample size than previous studies, and a longer follow-up period (until the age of 10 years). However, our study faced some limitations, which should be noted. Although we had nearly double the sample size than in the available studies in the United States on the subject (Dannemiller et al. 2014; Lynch et al. 2014), caution is warranted when interpreting the findings, due to the reduced statistical power in the adjusted regression models. Although the information on atopy as well as other atopy-related health outcomes such as asthma or allergic rhinitis are available within the LISAplus study, we were not able to perform regression analysis due to the limited sample size of our study. This also concerns the inclusion of other possible confounding factors assessed around birth, such as parental atopy or the presence of pets at home. Furthermore, we predicted trends in microbial diversity using the tRFLP method, whose results are reproducible and comparable to sequencing results if diversity measures are taken as relative values rather than absolute values (Orcutt et al. 2009). However, our analysis did not account for the phylogenetic relationships of bacteria or fungi within the samples. A sequencing project could now find out whether and how the trends in diversity translate into the occurrence of microbial taxa. Moreover, it is not yet entirely clear how the storage of dust samples over a period of several years might affect microbial DNA. Therefore, an unknown, not quantifiable storage effect might have biased the results of our study. Similarly, targeting DNA of microbial community members accounts for neither activity nor for viability of these microbes, although a viability assay for whole communities has been recently published (Weinmaier et al. 2015). In addition, only children for whom dust samples have been available and with follow-up information at 6 or at 10 years were included. Hence, we cannot exclude that the results might be biased through losses in follow-up. Compared with the total study population, there is a higher proportion of mothers with high education as well as a higher prevalence of parental allergy in our study group. Last, microbial diversity was measured at only one time point—shortly after birth—and it is not clear whether the observed inverse effects can be exclusively attributed to the crucial period shortly after birth or whether a constant signal of exposure to microbial diversity is required. With increasing age, activities conducted in different places (e.g., kindergarten, school) might start to become more important, and the daily individual microbial exposure may change in composition. That might explain why the inverse effects were attenuated later in childhood and poses the question whether a constant signal of exposure to microbial diversity is required.

## Conclusion

Our study is an important contribution to the field of the urban dust microbiome in relation to atopic and respiratory health. We observed a significant reduced risk for developing specific allergic sensitization to aeroallergens in early childhood after exposure to higher fungal diversity around birth, but with attenuated effects until later childhood. Further research is advised to regularly monitor microbial diversity throughout childhood and to identify key environmental characteristics capable of creating a microbial environment beneficial for allergic and respiratory health.

## Supplemental Material

(142 KB) PDF
