# Supplementary material for: Urban Dust Microbiome: Impact on Later Atopy and Wheezing
Source: Environ Health Perspect. 2016 May 27;124(12):1919–23. doi: 10.1289/EHP158 (PMC5132631; doi:10.1289/EHP158)
Supplement: (142 KB) PDF [file EHP158.s001.acco.pdf]

Environ Health Perspect
DOI: 10.1289/EHP158

Note to readers with disabilities: *EHP* strives to ensure that all journal content is accessible to all readers. However, some figures and Supplemental Material published in *EHP* articles may not conform to 508 standards due to the complexity of the information being presented. If you need assistance accessing journal content, please contact ehp508@niehs.nih.gov. Our staff will work with you to assess and meet your accessibility needs within 3 working days.

Supplemental Material

Urban Dust Microbiome: Impact on Later Atopy and Wheezing

Christina Tischer, Fabian Weikl, Alexander J. Probst, Marie Standl, Joachim Heinrich, and Karin Pritsch

Table of Contents

Table S1: Adjusted* odds ratios (aORs 95% CI) for the association between fungal and bacterial diversity (Simpson index, tertiles) and early wheezing (up to 2 years of age)

Table S1: Adjusted* odds ratios (aORs 95% CI) for the association between fungal and bacterial diversity (Simpson index, tertiles) and early wheezing (up to 2 years of age)

**Logistic regression models**	**Fungal diversity**	**Bacterial diversity**
**Wheezing ever (2y)**		
2nd tertile vs 1st tertile	0.58 (0.27, 1.23)	0.75 (0.35, 1.62)
3rd tertile vs 1st tertile	**0.36 (0.16, 0.83)**	0.97 (0.45, 2.08)
**GEE model** (longitudinal analysis)		
**Wheezing until 2y**		
2nd tertile vs 1st tertile	0.56 (0.29, 1.06)	0.61 (0.30, 1.21)
3rd tertile vs 1st tertile	**0.40 (0.18, 0.89)**	1.11 (0.57, 2.15)

*adjusted for sex, maternal education, season of dust sampling
